# Ubiquitin Ligase SMURF2 Interacts with Filovirus VP40 and Promotes Egress of VP40 VLPs

**DOI:** 10.3390/v13020288

**Published:** 2021-02-12

**Authors:** Ariel Shepley-McTaggart, Michael Patrick Schwoerer, Cari A. Sagum, Mark T. Bedford, Chaitanya K. Jaladanki, Hao Fan, Joel Cassel, Ronald N. Harty

**Affiliations:** 1 Department of Pathobiology, School of Veterinary Medicine, University of Pennsylvania, Philadelphia, PA 19104, USA; relshepley@gmail.com (A.S.-M.); mps3@princeton.edu (M.P.S.); 2 Department of Epigenetics & Molecular Carcinogenesis, M.D. Anderson Cancer Center, University of Texas, Smithville, TX 78712, USA; CASagum@mdanderson.org (C.A.S.); mtbedford@mdanderson.org (M.T.B.); 3 Bioinformatics Institute, Agency for Science, Technology and Research (A*STAR), 30 Biopolis Street, Matrix #07-01, Singapore 138671, Singapore; chaitanyakj@bii.a-star.edu.sg (C.K.J.); fanh@bii.a-star.edu.sg (H.F.); 4 Synthetic Biology Translational Research Programme, School of Medicine, National University of Singapore, Singapore 119077, Singapore; 5 Programme in Cancer & Stem Cell Biology, DUKE-NUS Medical School, Singapore 169857, Singapore; 6 Molecular Screening Facility, The Wistar Institute, Philadelphia, PA 19104, USA; jcassel@Wistar.org

**Keywords:** filovirus, Ebola, Marburg, VP40, SMURF2, E3 ubiquitin ligase, PPxY motif, WW-domain, VLP budding

## Abstract

Filoviruses Ebola (EBOV) and Marburg (MARV) are devastating high-priority pathogens capable of causing explosive outbreaks with high human mortality rates. The matrix proteins of EBOV and MARV, as well as eVP40 and mVP40, respectively, are the key viral proteins that drive virus assembly and egress and can bud independently from cells in the form of virus-like particles (VLPs). The matrix proteins utilize proline-rich Late (L) domain motifs (e.g., PPxY) to hijack specific host proteins that contain WW domains, such as the HECT family E3 ligases, to facilitate the last step of virus–cell separation. We identified E3 ubiquitin ligase Smad Ubiquitin Regulatory Factor 2 (SMURF2) as a novel interactor with VP40 that positively regulates VP40 VLP release. Our results show that eVP40 and mVP40 interact with the three WW domains of SMURF2 via their PPxY motifs. We provide evidence that the eVP40–SMURF2 interaction is functional as the expression of SMURF2 positively regulates VLP egress, while siRNA knockdown of endogenous SMURF2 decreases VLP budding compared to controls. In sum, our identification of novel interactor SMURF2 adds to the growing list of identified host proteins that can regulate PPxY-mediated egress of VP40 VLPs. A more comprehensive understanding of the modular interplay between filovirus VP40 and host proteins may lead to the development of new therapies to combat these deadly infections.

## 1. Introduction

Ebola (EBOV) and Marburg (MARV) viruses are members of the family *Filoviridae* and the causative agents of severe hemorrhagic fever associated with high mortality rates in humans. Both viruses are categorized by the Centers for Disease Control and Prevention and the National Institutes of Health (NIH) as BSL-4 agents. Currently, there are no approved therapeutic treatments available for these devastating diseases, leaving supportive and symptomatic care as the existing treatment options [1,2]. Therefore, there is a vital need for the development of effective and safe therapeutics against EBOV and MARV. A better understanding of the interplay between the virus and host will provide valuable insight toward the identification of novel therapeutic targets to combat these deadly infections.

The negative stranded, non-segmented RNA genome replicates in the cytoplasm, while assembly and budding of viral particles at the plasma membrane is mediated by the VP40 matrix protein. VP40 matrix proteins are similar in function to the matrix (M) protein of rhabdoviruses and paramyxoviruses, as well as the gag polyprotein of retroviruses, as they are all necessary and sufficient to associate with the plasma membrane and bud independently as noninfectious virus-like particles (VLPs) from mammalian cells [3,4,5,6,7,8,9,10]. Efficient budding of VLPs is dependent, in part, on conserved amino acid motifs within the matrix protein called late budding domains (L-domains) that include proline-rich PPxY and PTAP motifs (where “x” is any amino acid). For example, filoviruses utilize their VP40-encoded PPxY motifs to hijack specific WW domains containing host proteins to regulate the budding process and facilitate efficient virus–cell separation [4,11,12,13,14,15]. More specifically, we and others have shown that the PPxY motif of EBOV and MARV VP40 interact with the WW domains of HECT family E3 ubiquitin ligases Nedd4, ITCH, and WWP1, leading to mono-ubiquitinylation of the viral matrix proteins and facilitation of VLP egress. It is thought that the PPxY/WW domain interactions between VP40 and host E3 ligases provide a functional link between VP40 and the host ESCRT (endosomal sorting complex required for transport) machinery to facilitate virus–cell separation at the plasma membrane [4,10,11,12,13,14,15,16,17]. We are interested in identifying the full repertoire of HECT family E3 ligases that can interact with and promote VP40 VLP egress, as this will be important for understanding the overall virus–host interface and may lead to the identification of a promising drug target(s) for the development of novel therapeutics.

Toward that end, we used an unbiased protein–protein interaction screen whereby biotinylated eVP40, mVP40 PPxY-containing wildtype, or mutant peptides were incubated with an array of approximately 115 mammalian WW domains expressed as GST-fusion proteins to identify novel host interactors. Results from this screen demonstrated that the VP40 PPxY motifs bound specifically to a select number of host WW domain-bearing proteins, while the PPxY mutant peptides did not bind to any of the host proteins on the array. Here, for the first time, we reported on the identification of HECT family E3 ligase SMURF2 (Smad Ubiquitin Regulatory Factor 2) as a VP40 interactor. Originally identified as a negative regulator of the BMP and TGFβ signaling pathways, SMURF2 plays role in embryogenesis, homeostasis, and cellular differentiation, as well as carcinogenesis and human disease pathogenesis [18,19,20,21]. We go on to show by GST pulldown assays that the multiple WW domains of SMURF2 interact with eVP40 and mVP40 in a PPxY-dependent manner. In addition, protein–peptide docking analysis and results from a surface plasmon resonance assay support and confirm this newly identified virus–host PPxY/WW domain interaction. Using eVP40 as our model, we demonstrated that siRNA knockdown of endogenous SMURF2 led to inhibition of eVP40 VLP egress, suggesting that the interaction between eVP40 and SMURF2 is functional by positively regulating eVP40 VLP budding. Together, our results indicate that host E3 ligase SMURF2 physically interacts with filoviral matrix proteins eVP40 and mVP40 in a PPxY/WW domain-dependent manner to positively regulate VLP budding. These findings provide further and consistent evidence for the important role of a broad array of host WW domain-containing HECT family E3 ligases in positively regulating PPxY-mediated egress of filovirus VP40 VLPs.

## 2. Materials and Methods

### 2.1. Cell Lines, Plasmids, and Reagents

HEK293T were maintained in Dulbecco’s (Radnor, PA, USA) modified Eagle’s medium (DMEM) supplemented with 10% fetal calf serum (FCS) and penicillin (100 U/mL)–streptomycin (100 μg/mL) at 37 °C in a humidified 5% CO_2_ incubator. Plasmids expressing wild-type eVP40 (eVP40-WT) and eVP40-ΔPT/PY (PTAPPEY deletion mutant) were described previously [20,22]. Plasmids expressing FLAG-tagged mVP40-WT and PPxY mutant mVP40 (*P* > A) were kindly provided by S. Becker (Institut fur Virologie, Marburg, Germany). Plasmids expressing SMURF2-FLAG-WT or the enzymatically inactive SMURF2-FLAG-C716A mutant were purchased from Addgene (Watertown, MA, USA, catalog numbers 11746 and 11747). SMURF2-specific or random small interfering RNAs (siRNAs) were purchased from Dharmacon (Lafayette, CO, USA, catalog number L-007194-00-0005 and D-001810-10-20). Mouse anti-FLAG monoclonal antibody was purchased from Fitzgerald Industries International (Acton, MA, USA, catalog number 10R-2139), rabbit anti-SMURF2 was purchased from Cell Signaling Technology (Danvers, MA, USA, catalog number 12024S), and mouse anti-GST (catalog number SAB4200237) as well as mouse anti-β-actin (catalog number A1978) antisera were obtained from Sigma-Aldrich (St. Louis, MO, USA). Rabbit anti-eVP40 antibody was purchased from IBT Bioservices (Rockville, MD, USA, catalog number 0301-010).

### 2.2. Protein Array Experiments

To generate the protein-domain reading array, the WW and SH3 domains were codon optimized for bacterial expression and cloned into a pGex vector. All WW and SH3 domains were expressed as GST fusions in *Escherichia coli* and purified on glutathione–sepharose beads. The recombinant domains were arrayed onto nitrocellulose-coated glass slides (OncyteAvid slides; Grace Bio-Labs, Bend, OR, USA) using an Aushon 2470 arrayer with solid pins as described previously [23]. Fluorescence labeling of the biotinylated peptide probe and slide binding were also described previously [23]. Four peptides were tested on the array: eVP40-WT (MRRVILPTAPPEYMEAI-K-biotin), eVP40-Mut (MRRVILPTAAAEAMEAI-K-biotin), mVP40-WT (MQYLNPPPYADHGGANQL-K-biotin), and mVP40-Mut (MQYLNAAPAADHGANQL-K-biotin). The fluorescent signal was detected by using a GeneTac LSIV scanner (Genomic Solutions, Ann Arbor, MI, USA).

### 2.3. Docking Analysis

The protein–peptide docking analysis was employed using the Glide module of Schrödinger [24]. The SMURF2 WW3 domain was obtained from PDB (PDB ID: 2LTZ) and the structure was prepared using the Protein Preparation Wizard tool. The eVP40 peptide (MRRVILPTAPPEYMEAI), mVP40 peptide (MQYLNPPPYADHGANQL), SMAD7 peptide (ELESPPPPYSRYPMDFL, positive control), and mutant peptide (MRRVILPTAAEAMEAI, negative control) were built with Maestro and generated multiple conformers using the MacroModel sampling method [25]. The receptor grid for peptide docking purposes was generated with default settings and centroid of the SMAD7 peptide (B chain of 2LTZ) defined as grid center. The Glide SP-PEP protocol was used to dock peptide conformers [24].

### 2.4. Expression and Purification of GST Fusion Proteins

GST-WW domain fusion proteins were purified from *E. coli* BL21(DE3) cells grown in LB broth with appropriate antibiotics at 37 °C. GST-WW domain fusion proteins were induced with isopropyl-β-d-thiogalactopyranoside (IPTG) (0.2 mM) for 4 h at 30 °C. Bacterial cultures were centrifuged at 5000 rpm for 10 min at 4 °C, and lysates were extracted by using B-PER bacterial protein extract reagent according to the protocol supplied by the manufacturer (Thermo Fisher Scientific, Philadelphia, PA, USA). GST-WW domain fusion proteins were purified with glutathione–sepharose 4B and eluted with elution buffer (100 mM Tris-Cl (pH 8.0), 120 mM NaCl, 30 mM reduced glutathione). Purified proteins were analyzed on SDS-PAGE gels and stained with Coomassie blue.

### 2.5. GST-Pulldown Assay

HEK293T cells were transfected with either eVP40-WT, eVP40-Mut, mVP40-WT, or mVP40-Mut. At 24 h after transfection, the cell extracts were incubated with the GSH beads described above at 4 °C for 4 h with continuous rotating. The protein complexes were pulled down with beads via centrifugation. The rabbit eVP40 antiserum (IBT Bioservices, Rockville, MD, USA), mouse anti-flag monoclonal antibody (Fitzgerald Industries International, Acton, MA, USA), and mouse anti-GST monoclonal antibody (Sigma-Aldrich, St. Louis, MO, USA) were used to detect eVP40-WT, eVP40-ΔPT/PY, mVP40, mVP40 mutant, or GST-SMURF2 WW proteins in input and pulldown samples by Western blotting.

### 2.6. Surface Plasmon Resonance

Binding of both wild-type and mutant eVP40 peptides to SMURF2 was assessed by surface plasmon resonance (SPR) using a Biacore T200 instrument. SMURF2 was immobilized on a carboxymethyldextran sensor chip using standard amine coupling procedures. Each peptide was serially diluted 1:2 in running buffer (20 mM HEPES, pH 7.4, 150 mM NaCl, 0.05% Tween 20) starting at 200 uM. The association time was 120 s, the dissociation time was 300 s, and the flow rate was 30 uL/min. Due to the fast kinetics of the peptides binding to SMURF2, no regeneration conditions were used between injections. The affinities of both peptides were determined by one-site fits to the equilibrium binding plots using the Biacore Evaluation software.

### 2.7. VLP Budding Assays

Filovirus VLP budding assays using HEK293T cells and eVP40 were described previously [10,11,16,26,27]. eVP40 proteins in VLPs and cell extracts were detected by SDS-PAGE and Western blotting and quantified using NIH Image-J software. The anti-eVP40 antiserum was used to detect eVP40-WT, and anti-FLAG monoclonal antibody was used to detect FLAG-tagged SMURF2.

### 2.8. siRNA Knockdown Assay

HEK293T cells in Opti-MEM in collagen-coated 6-well plates were transfected twice with either control siRNAs or SMURF2-specific siRNAs at a final concentration of 100 nM by using Lipofectamine (Invitrogen, Waltham, MA, USA) at 2-day intervals. A total of 0.5 μg of eVP40 and/or SMURF2-FLAG plasmid DNA was transfected with the second round of siRNAs. Cell extracts and VLPs were harvested at 24 h post-transfection and the indicated proteins were detected in cell and VLP samples by Western blotting using specific antisera.

## 3. Results

### 3.1. Identification of VP40 WW Domain Interactors

**Figure 1:**
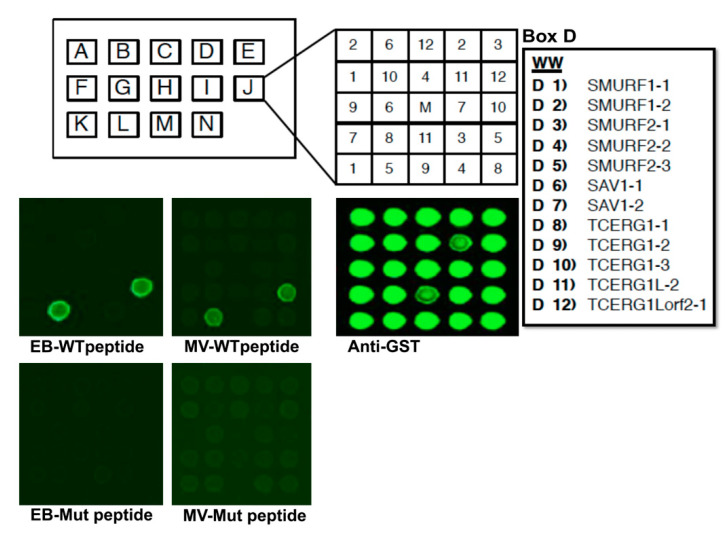
WW domain array screen. Purified GST/WW domain fusion proteins were arrayed in duplicate in Boxes A–N in numbered squares 1–12 as shown. The GST/WW domain fusion proteins present in Box D are shown. The array was screened with the following biotin-labeled peptides: EB-WT (MRRVILPTAPPEYMEAI-K-biotin), EB-Mut (MRRVILPTAAAEAMEAI-K-biotin), MV-WT (MQYLNPPPYADHGGANQL-K-biotin), or MV-Mut (MQYLNAAPAADHGANQL-K-biotin). A positive interaction was observed between SMURF2-3 (position #5) and both EB-WT and MV-WT peptides. An anti-GST control for protein expression is shown. The center M square contains GST alone.

To identify new host WW domain-containing proteins that interact with the PPxY motifs of eVP40 and mVP40, we used WT and PPxY mutant peptides of eVP40 and mVP40 to probe ~115 known WW domains. The biotinylated peptides were fluorescently labeled and used to screen a specially prepared proline-rich reading array composed of almost all known WW domain-containing proteins, as well as 40 SH3 domain-containing proteins (Figure 1). We show that both eVP40-WT and mVP40-WT peptides interacted with a select number of host WW domains indicated by green fluorescence, whereas the PPxY mutant peptides did not interact with any of the host WW domains on the array. As expected from our prior studies, WW domains from E3 ubiquitin ligases Nedd4, ITCH, and WWP1 [4,11,12,13,14,15] were identified as positive interactors. Interestingly and for the first time, we also observed an interaction between both WT VP40 peptides and WW domain #3 from the HECT family E3 ubiquitin ligase SMURF2 (Smad Ubiquitin Regulatory Factor 2) (Figure 1). The select binding of WT, but not PPxY mutant peptides to SMURF2 WW domains suggests that this virus–host interaction may play a role in regulating VP40-mediated egress.

### 3.2. Protein–Peptide Docking Analysis Supports VP40 PPxY Binding to WW3 of SMURF2

**Figure 2:**
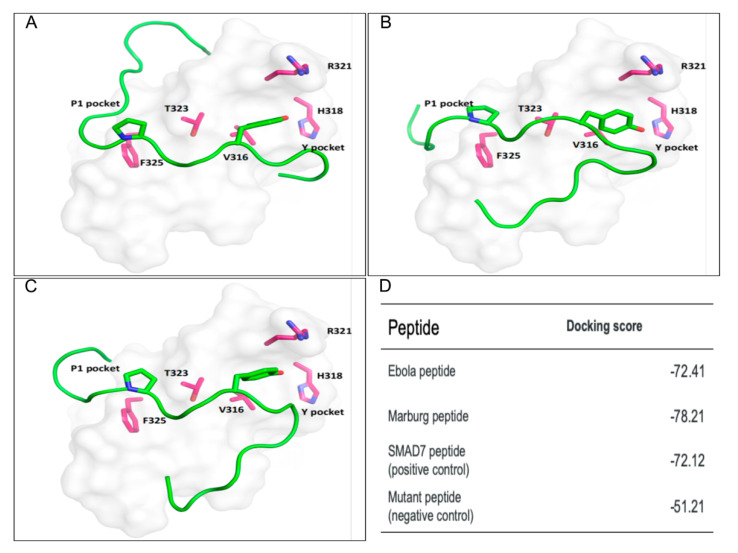
Docking Models of PPxY/WW interactions. Docking models showing PPxY/WW interactions for: (**A**) eVP40-WT, (**B**) mVP40-WT, and (**C**) SMAD7 PPxY-containing peptides with WW domain #3 from SMURF2. (**D**) Protein–peptide docking scores.

To support our finding of a possible interaction between the PPxY motifs of eVP40, mVP40, and the third WW domain of SMURF2, we performed docking analysis to model the VP40 PPxY/SMURF2 WW domain interface and assess the likelihood of this interaction occurring in infected cells. As a positive control for binding to the SMURF2 WW domain we used a PPxY peptide derived from SMAD7, a known biologically relevant SMURF2 binding partner [28]. The structure of WW3 domain from SMURF2 was obtained from PDB (PDB ID: 2LTZ), and we used the Glide module of Schrödinger’s to dock the eVP40 (Figure 2A) and mVP40 peptides (Figure 2B), as well as SMAD7 peptide (Figure 2C) to this WW domain. Modeling of the peptides (shown in green) as ligands for the modular SMURF2 WW3 domain (in grey) is shown with eVP40 peptide (Figure 2A) and mVP40 peptide (Figure 2B). Our results revealed that the mVP40 PPxY peptide had the best docking score compared to that of the eVP40 PPxY peptide and the positive control SMAD7 peptide. Moreover, the eVP40 peptide showed a comparable binding score to that of the SMAD7 peptide (Figure 2D).

The binding site of SMURF2 WW3 domain is a non-canonical WW domain with an F in place of the canonical binding site W at position 325. The peptide docking analysis against the SMURF2 WW3 domain showed that the P1 of the PPxY motif fits in the P1 pocket which is formed by Y314, T323, and F325 (Figure 2A–C), the P1 and P2 of the PPxY motif stacked with F325 and Y314, respectively. The carbonyl backbone of the P2 of the PPxY motif interacts with T323 with a hydrogen bond as seen in other WW domain interactions. The Y sidechain of the PPxY motif occupies the Y pocket which is a hydrophobic groove consisting of sidechains from V316, H318, R321, and T323 (Figure 2A–C). This indicates that although canonical binding site W is replaced by F at position 325, the PPxY peptide binds to the SMURF2 WW3 domain with a similar orientation to that observed for interactions between other PPxY peptides and canonical group I WW domains. Additionally, in the complex of the mVP40 peptide and SMURF2 WW3 domain, the Q (P1−4 position) and Y (P1−3 position) residues before the PPxY motif form hydrogen-bonding interactions with D327 and R329, and the histidine residue of the mVP40 peptide formed a salt bridge interaction with E304 of the SMURF2 WW3 domain, respectively (Figure 2B). Those interactions were not observed with the other peptides. These results support the potential for a VP40–SMURF2 interaction to occur during virus infection and thus could play a role in regulating VP40-mediated budding.

### 3.3. Surface Plasmon Resonance Confirms the Modular VP40 PPxY/SMURF2 WW Domain Interaction

**Figure 3:**
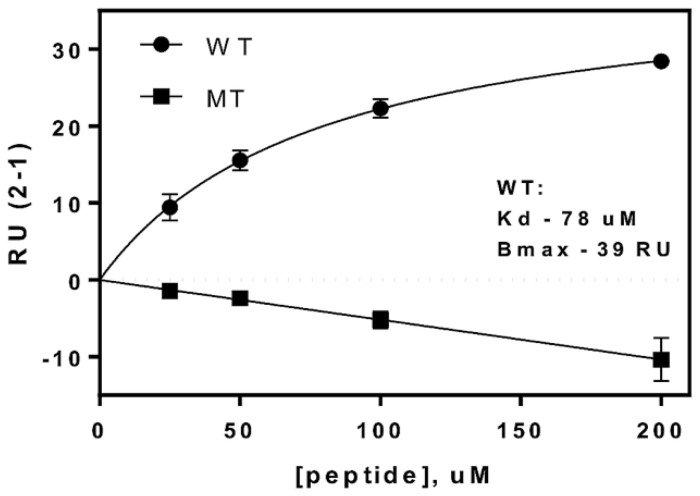
Surface plasmon resonance. Graphical evaluation of eVP40-WT and PPxY mutant peptides binding to GST/WW domain #3 of SMURF2 with response units (RU) plotted on the *Y*-axis and peptide concentration in uM plotted on the *X*-axis. The Kd for the WT peptide was calculated to be 78 uM with a Bmax of 39 RU.

Here we sought to confirm the observed interaction between the VP40 PPxY motif and WW domain #3 of SMURF2 using a highly sensitive surface plasmon resonance assay. Briefly, purified GST-SMURF2-WW3 was immobilized on the sensor chip and serially diluted samples of eVP40-WT and eVP40 PPxY mutant peptides were flowed across the chip to measure their association kinetics (Figure 3). We found that eVP40-WT peptide bound robustly to WW3 of SMURF2 in a concentration-dependent manner, whereas the PPxY mutant peptide did not associate with WW3 at any concentration tested (Figure 3). These results correlate well with those described above and indicate that the peptide-expressed PPxY motif of VP40 can interact with modular WW domain #3 from SMURF2.

### 3.4. GST Pulldown Assays to Assess Binding of VP40 PPxY Motifs to SMURF2 WW Domains

**Figure 4:**
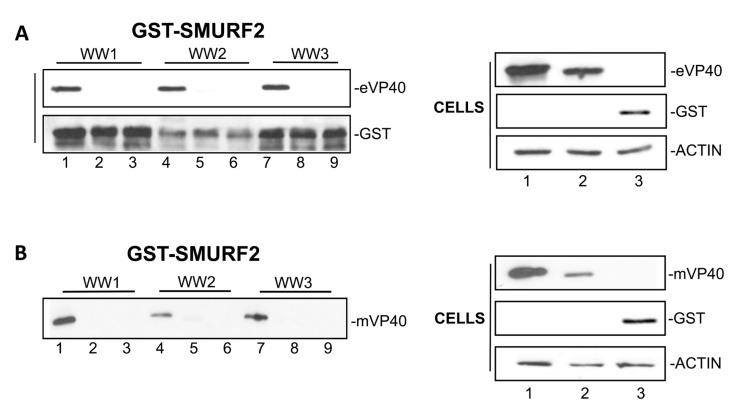
GST pulldown between SMURF2 WW domains and full length VP40. (**A**) GST pulldown assay using purified GST-SMURF2 WW domains 1, 2, and 3 with HEK293T cell extracts containing eVP40 WT (lanes 1, 4, and 7), eVP40 PPxY mutant (lanes 2, 5, and 8), or GST alone (lanes 3, 6, and 9). (**B**) GST pulldown assay using purified GST-SMURF2 WW domains 1, 2, and 3 with HEK293T cell extracts containing mVP40 WT (lanes 1, 4, and 7), mVP40 PPxY mutant (lanes 2, 5, and 8), or GST alone (lanes 3, 6, and 9). Input levels of eVP40, mVP40, GST, and actin are shown.

Here we used purified GST-WW domain fusion proteins and GST pulldown assays to confirm the specific PPxY/WW interaction between full-length VP40 and SMURF2. Interestingly, we found that full-length eVP40-WT and mVP40-WT, but not the full-length PPxY mutants, expressed exogenously in HEK293T cells, interacted with all three WW domains from SMURF2 (Figure 4). Of the three SMURF2 WW domains, WW domain #3 appeared to interact with eVP40 and mVP40 more strongly than WW domains #1 and #2 (Figure 4), which is consistent with the results observed in the WW domain screening array (Figure 1). These data also suggest that the PPxY motifs expressed in the context of full-length VP40 are capable of interacting with multiple WW domains of SMURF2. This finding is not surprising since the VP40 PPxY motifs have previously been shown to interact with multiple WW domains from other mammalian E3 ligases such as Nedd4, WWP1, and ITCH, albeit with varying degrees of strength [11,13,14,15].

### 3.5. Exogenously Expressed SMURF2 Enhances Egress of eVP40 VLPs

**Figure 5:**
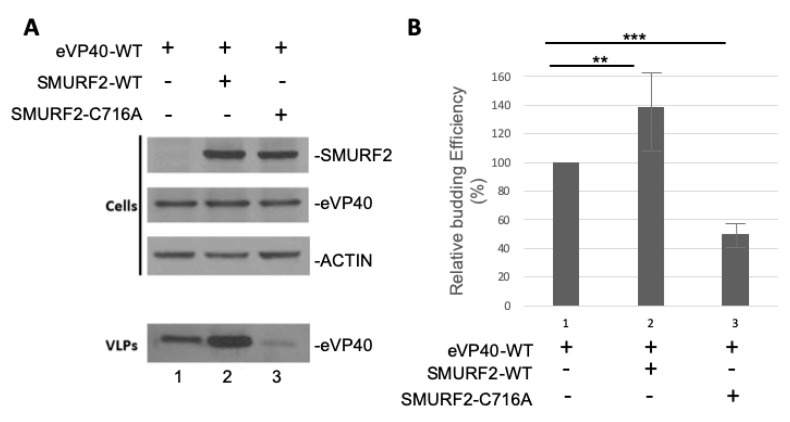
Enzymatically active SMURF2 positively regulates eVP40 VLP budding. (**A**) Representative Western blot of HEK293T cell extracts and VLPs of eVP40 alone (lane 1), eVP40 + SMURF2-WT (lane 2), and eVP40 + SMURF2-C716A mutant (lane 3). (**B**) Graph showing the relative budding efficiency of eVP40 VLPs under the indicated conditions from three independent experiments; student t test, ** = *p* <0.01, *** = *p* <0.005.

To investigate whether the eVP40/SMURF2 interaction was functional in regulating budding of VP40 VLPs as reported for other HECT family E3 ligases [4,11,12,13,14,15], we used our well-established VP40 VLP budding assay. Briefly, we transfected HEK293T cells with eVP40 alone or with equivalent amounts of either SMURF2-WT or the catalytically inactive mutant SMURF2-C716A. Cell extracts and VLPs were harvested and the indicated proteins were detected by Western blotting (Figure 5A). We observed a modest increase in eVP40 VLP budding in the presence of SMURF2-WT compared to eVP40 alone; however, we did not observe any increase in eVP40 VLP budding in the presence of SMURF2-C716A (Figure 5B graph is representative of three independent experiments) as compared to eVP40 alone. Excitingly, expression of SMURF2-C176A appeared to have a dominant negative effect on eVP40 VLP budding as the budding efficiency was reduced significantly in the presence of the catalytically inactive SMURF2-C176A mutant as compared to controls (Figure 5B). These data indicate that the enzymatic activity of SMURF2 is required to positively regulate eVP40 VLP budding.

### 3.6. Expression of Endogenous SMURF2 Is Important for Efficient Egress of eVP40 VLPs

**Figure 6:**
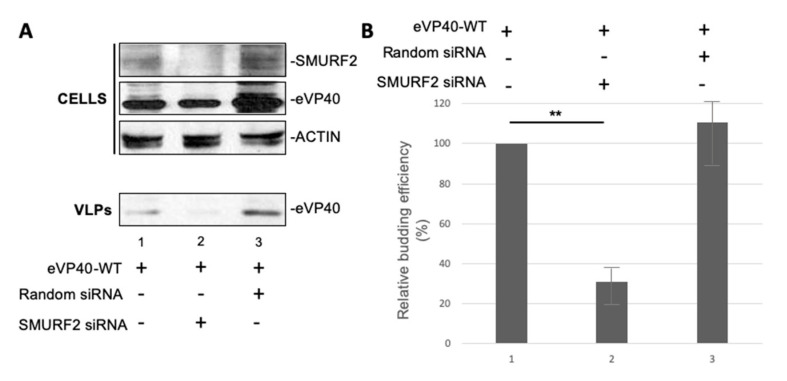
SiRNA knockdown of SMURF2 decreases VP40 VLP budding efficiency. (**A**) HEK293T cells were transfected as indicated and the indicated proteins were detected in cell extracts and VLPs by Western blotting. (**B**) Graphical representation of the relative budding efficiency of eVP40 under the indicated conditions from three independent experiments; student t test, ** = *p* <0.005.

To further assess the biological relevance of a SMURF2/VP40 interaction in positively regulating egress of VP40 VLPs we used an siRNA knockdown approach. Briefly, HEK293T cells were mock transfected or transfected with eVP40-WT plus nonspecific or SMURF2-specific siRNAs. Cell extracts and VLPs were collected and expression levels of eVP40-WT and endogenous SMURF2 were observed by Western blotting (Figure 6A). SMURF2-specific siRNAs efficiently knocked down expression of endogenous SMURF2 by >75% (Figure 6A, lane 2) and we observed a concomitant decrease of approximately 80% in the levels of eVP40 VLPs (Figure 6B, lane 2). Although there was a slight increase in VP40 budding of eVP40 VLPs in the presence of nonspecific siRNA, this difference was statistically insignificant as compared to expression of eVP40 alone (Figure 6B graph is representative of three independent experiments). These findings provide strong evidence that endogenously expressed SMURF2 in HEK293T cells is important for efficient egress of eVP40 VLPs and that SMURF2 can be included in the repertoire of mammalian HECT family E3 ubiquitin ligases that can function to positively regulate VP40-mediated egress of VLPs.

## 4. Discussion

In mammalian cells, modular interactions between WW domains and PPxY-containing proteins regulate many pathways, including protein ubiquitination, signaling, sorting, migration, and degradation processes [11,20,29,30,31,32]. Several viruses, including the filoviruses EBOV and MARV, have evolved the ability to mimic these protein–protein interactions by using virally encoded PPxY-containing proteins to bind to specific host proteins bearing one or more WW domains, thus, in essence, hijacking cellular pathways and networks that ultimately impact both the virus and the host. Indeed, previous studies have shown that the filoviral matrix proteins eVP40 and mVP40 contain PPxY motifs that interact with WW domain-containing HECT family E3 ligases to facilitate budding from the plasma membrane [4,11,12,13,14,15,17]. Here, we report for the first time on the identification of HECT family member SMURF2 as the newest WW domain-containing E3 ligase that can interact physically and functionally with PPxY-containing filovirus VP40 proteins.

A specific physical interaction between the viral PPxY motifs and WW domains of SMURF2 were confirmed using both GST pulldown (Figure 4) and Surface Plasmon Resonance assays (Figure 3), as well as in silico peptide/protein docking analysis (Figure 2). In addition to the observed physical interaction between VP40 and SMURF2, we demonstrated that this interaction was functional as well in our VLP budding assay. Our results showed that exogenous expression of WT SMURF2, but not the enzymatically inactive C>A mutant of SMURF2, resulted in a significant increase in the relative budding efficiency of VP40 VLPs (Figure 5). These results were then confirmed using an siRNA knockdown approach which showed that expression of endogenous SMURF2 was important for efficient VP40 VLP egress (Figure 6). To our knowledge, this is the first description of a functional interaction between SMURF2 and filoviral VP40 proteins.

SMURF2 is one of nine members of the HECT family of E3 ubiquitin ligases, with Nedd4 being the prototypical ligase in the family. Interestingly, expression of SMURF proteins is increased in several epithelial and endothelial cell types in which filoviruses replicate robustly, including gastrointestinal endothelial cells, placental villi epithelial cells, and epithelial cells of the testes [18,19,20,21,33,34]. We speculate that it would be beneficial for VP40 to interact with additional HECT family E3 ligases, like SMURF2, that are present in relatively high abundance during infection of those epithelial or endothelial cell types. Although it is likely that the expression levels of these numerous HECT family E3 ligases will vary depending on the cell type, their function in positively regulating VP40-mediated egress is likely to be consistent across cell types. Additionally, SMURF2 has been previously indicated to be proviral in studies that showed the SMURF2 interaction with the anti-viral VISA protein led to its degradation and to the suppression of virus-triggered Type 1 IFN signaling [35]. The suppression of innate immunity as well as positive regulation of egress by SMURF2 could advance EBOV and MARV infectivity in cells types with relatively high expression of this host interactor. Also of note is that docking analysis of WW3 of SMURF2 with the VP40 PPxY peptides (Figure 2D) indicated that the mVP40 peptide had a better docking score as compared to that of the eVP40 peptide and the positive control SMAD7 peptide. Since SMAD7 acts as an adaptor protein and binds to SMURF2 and the TGF-β receptor to facilitate its degradation [21,28,36], it is tempting to speculate that the PPxY motif of mVP40 may compete with that of SMAD7 in MARV-infected cells for binding to SMURF2, which could affect host TGF-β signaling. Interestingly, EBOV infection of hepatocytes results in upregulation of TGF-β signaling and cellular markers of epithelial mesenchymal transition (EMT) [37]. Further investigation into how a potential SMURF2–VP40 interaction may regulate EMT and TGF-β secretion in EBOV or MARV infected cells is warranted.

While VLP budding assays are invaluable BSL-2 approaches to study the mechanisms and host interactions involved in EBOV and MARV egress, a role for SMURF2 in budding of authentic filoviruses remains to be determined. A better understanding of the interplay between filovirus and host factors will be critical for the overall understanding of the biology and pathogenesis of the viruses, as well as for the future development of effective antiviral therapies. Indeed, a growing body of literature has demonstrated that that PPxY/WW domain interactions play key roles in regulating one or more steps in the lifecycles of other viruses as well [17,38,39,40,41,42,43,44]. Inhibition of the viral–host PPxY/WW interaction may impede infection rates or reduce disease pathology of viral pathogens, including the filoviruses. Importantly, the results from this study support the possibility of the viral–host PPxY/WW domain interface serving as a viable target for the development of small-molecule inhibitors of virus budding as broad-spectrum antiviral therapeutics [45,46,47].

## Data Availability

All data are contained within the article.
